# Incidence of Atypical Femoral Fracture and Its Mortality in a Single Center in Singapore

**DOI:** 10.1002/jbm4.10515

**Published:** 2021-06-19

**Authors:** Linsey Gani, Natasha Anthony, Lily Dacay, Pei Tan, Le Roy Chong, Thomas FJ King

**Affiliations:** ^1^ Department of Endocrinology Department of Medicine Changi General Hospital 2 Simei Street 3 529889 Singapore Singapore; ^2^ Centre of Trial Research Unit Changi General Hospital Singapore Singapore; ^3^ Department of Radiology Changi General Hospital Singapore Singapore

**Keywords:** OSTEOPOROSIS, BISPHOSPHONATE, ATYPICAL FEMORAL FRACTURES, ASIAN, MORTALITY

## Abstract

Bisphosphonates (BP) are the most commonly prescribed effective form of osteoporosis treatment with adverse effects associated with prolonged use such as atypical femoral fractures (AFF). Asians have an elevated risk of AFF at 5 to 6 times those of whites and Hispanics. In this study, we characterize factors associated with AFF and its mortality in a single center in Singapore. We conducted a cohort study of subjects older than 50 years admitted to Changi General Hospital (CGH), Singapore, with fragility subtrochanteric femoral fractures from 2009 to 2015. Using the ASBMR 2014 criteria, fractures are classified into atypical and typical subtrochanteric femoral fractures. CGH uses a nationalized electronic health record that allows review of information on patients' demographics, clinical history and previous investigations. Mortality was assessed as of December 31, 2019. Between 2009 and 2015, there were 3097 hip fractures, of which 393 were subtrochanteric femoral fractures and 69 were classified as AFF by ASBMR 2014 criteria. A total of 52.2% of AFF occurred with BP exposure of median duration 56.5 (28 to 66) months. Multivariate regression showed that BP exposure was associated with the highest risk of AFF (odds ratio [OR] = 6.65 [2.35–18.9]). AFF patients had higher 5‐year survival (0.85 versus 0.62, *p* = 0.001) compared with typical subtrochanteric fracture patients. However, after adjusting for variables, the type of subtrochanteric femoral fractures were no longer significantly associated with progression to death, whereas older age, higher mean Charlson comorbidity score, and Malay ethnicity were the strongest predictors of death. AFF constitutes a small proportion of hip and femoral fractures with prolonged BP use being the highest risk factor for its development. There is no evidence of increased mortality or morbidity in patients with AFF compared with the typical subtrochanteric fracture. The fear of AFF should not impede treatment of typical osteoporotic fractures in this population. © 2021 The Authors. *JBMR Plus* published by Wiley Periodicals LLC on behalf of American Society for Bone and Mineral Research.

## 1 Introduction

Osteoporosis is a chronic degenerative disease that causes degradation of normal bone structure. It renders an individual to be at higher risk of major fractures. It is projected that more than 50% of all osteoporotic fractures will occur in Asia by the year 2050.^(^ ^1^ ^)^ Since their introduction in the 1990s, bisphosphonates (BP) have been the mainstay of osteoporosis treatment. BPs inhibit osteoclast‐mediated resorption and remodeling of bone.^(^ ^2^ ^)^ Many large randomized controlled trials have established the efficacy of BP, showing their ability to increase bone mineral density (BMD) and decrease the risk of hip and vertebral fractures by as much as 40% to 70%.^(^ ^3^ ^)^ In Singapore, oral BP is still the first line of treatment for osteoporosis for most patients. Its affordable cost and oral route of administration render the drug a viable option to most patients.

Case reports of unusual fragility fractures in the subtrochanteric region and along the femoral diaphysis in BP‐treated patients emerged in the literature about 15 years ago. Singapore was one of the first countries in the world to have reported these fractures.^(^ ^4^ ^)^ This was followed by larger studies of these fractures (now known as atypical femur fractures [AFF]) and their relation to BP.^(^ ^5^, ^6^, ^7^ ^)^ The pathogenesis of AFF is currently unclear; BP may alter intrinsic bone properties and healing of microcracks, leading to accumulation of microdamage and stress fracture. Other individual specific risk factors may increase susceptibility, either through alterations in bone geometry or microarchitecture, by interacting with antiresorptives, or by increasing biomechanical stress on the femur. These include ethnicity, younger age, higher body mass index (BMI), genetic factors, comorbidities, and concomitant drugs such as corticosteroid use.^(^ ^6^ ^)^ A recent large network analysis in California reported that Asian race formed half of their AFF cohorts; risk of AFF in Asians was 4.84 versus whites after adjusting for potential confounding variables.^(^ ^8^ ^)^ Another recent AFF study in Australia also found that among all Asian patients with AFF, Southeast Asian patients may display the highest risk.^(^ ^9^ ^)^

Although increased mortality associated with typical fragility fractures of the femur is known,^(^ ^10^, ^11^ ^)^ the rate of death associated with AFF has not been well established. A previous study in Sweden found lower mortality in AFF compared with ordinary fractures,^(12^ ^)^ whereas another study in an elderly population in UK found no difference in mortality at 30 days.^(^ ^13^ ^)^ No previous studies in the Southeast Asian population have been performed. Given the likely higher risk of AFF in this population, we set out to study the incidence of AFF in our Southeast Asian population and its related demographic and clinical risk factors. We also assessed mortality rate of patients who have sustained AFF versus those with typical subtrochanteric femoral fractures.

## 2 Materials and Methods

**Fig 1:**
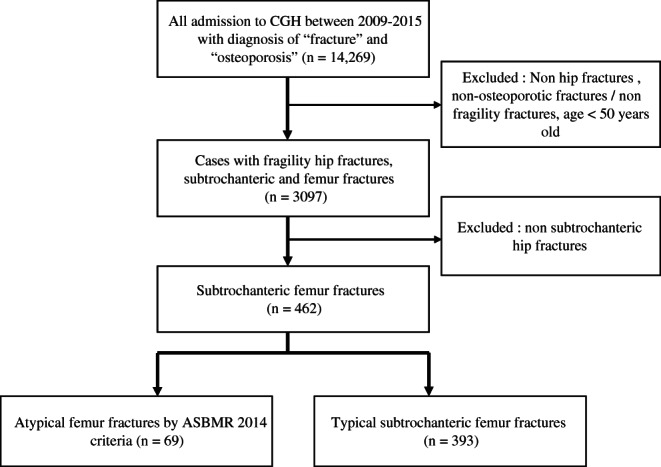
Flow chart of identification of atypical femoral fracture (AFF) and subtrochanteric femur fractures.

We conducted a cohort study of all patients admitted to Changi General Hospital (CGH) with acute fragility hip, subtrochanteric, and femur fractures from 2009 to 2015. CGH is a regional hospital serving the eastern population of Singapore estimated at 1.3 million. It employs a nationalized electronic health record that stores demographic information, past biochemical and radiological investigations, and prescription records, which can be accessed from all public health institutions in Singapore. All fragility fractures presenting to CGH between 2009 and 2015 were extracted from the electronic medical records with discharge diagnosis “fracture” and “osteoporosis.” Exclusion criteria include traumatic fractures (eg, motor vehicle car accident), and non‐osteoporotic or non‐fragility fracture and age younger than 50 years. We then separated all hip and subtrochanteric femur fractures and further extracted all subtrochanteric femoral fractures for further evaluation. Radiographs were obtained for patients with subtrochanteric or femoral shaft fractures to reclassify them into AFF according to the ASBMR 2014^(^ ^14^ ^)^ guideline for AFF (Fig. 1). Clinical and demographic data were extracted from the electronic medical records; this includes ethnicity data as listed in the patient's Singapore NRIC (National Registration Identity Card). Baseline clinical data were obtained on all patients at the time of presentation for fragility subtrochanteric and femoral shaft fractures. Information regarding medical history, including previous fragility fractures, parental history of fragility fractures, history of rheumatoid arthritis, current or previous steroid use in the last 12 months or prednisolone equivalent dose of 5 mg/d for >3 months at the time the fracture was recorded, and history of type 2 diabetes mellitus (DM2), was collected from the electronic medical records. Medication data collected include use of oral antidiabetic drugs, calcium and vitamin D supplementation, glucocorticoid use of more than 3 months’ duration, and anti‐osteoporosis drugs. Singapore has a national electronic health record that documents prescriptions across all public health institutions; duration of medication used was obtained from these prescription records. Duration of antiresorptive used was calculated as duration before the subtrochanteric femoral fractures. Antiresorptive use was analyzed if this was within 5 years of the fracture date and of continuous duration without a break of more than 6 months. Sequential treatment was observed in 15 patients, 11 patients had sequential oral BP (either alendronate or risedronate), and 4 patients were transitioned from oral BP to denosumab. Total exposures within 5 years were added together to constitute duration of BP exposure for each patient. There were no patients that were solely on denosumab for their documented antiresorptive treatment history. We further recorded data on smoking status and alcohol intake (>3 units/d). Smoking and alcohol intake were recorded if this was documented in the medical history before the fracture. Baseline anthropometric data were extracted from the electronic medical record and BMI was calculated as weight divided by height squared (kg/m^2^). All data were collected at time of presentation of the fracture.

### 2.1 Biochemical evaluation

Hemoglobin A1c (HbA1c) level (%) was determined by immunoturbidimetric assay (Cobas 8000, Roche Diagnostics, Basel, Switzerland). HbA1C values within 6 months of admitted date were included in this analysis. Serum creatinine (μmol/L) was measured using indirect ion‐specific electrode (Roche Diagnostics) and estimated glomerular filtration rate (eGFR) was calculated using the Chronic Kidney Disease Epidemiology (CKD‐EPI) Eq. 25‐hydroxyvitamin D (25‐OHD) was measured by radioimmunoassay (Roche Diagnostics), and thyroid‐stimulating hormone (TSH) levels were also measured by immunoassay (Abbot Affinity, Chicago, IL, USA). Baseline biochemical data were collected at the time of presentation for the fracture.

### 2.2 Radiological evaluation

All femoral radiographs performed at the time of patient presentation with acute fractures were retrospectively analyzed by adjudicating the radiographic findings with the reporting radiologist and our study member (CLR, a musculoskeletal radiologist with 20 years' experience), who was blinded to patients' identity and clinical characteristics, and classified according to the ASBMR 2014 criteria to ascertain if the radiographic appearances fulfill the definitions of AFF. Periprosthetic fractures were excluded from AFF cases. Healing of fracture was assessed through electronic records to the first documented radiographic report of callus formation or union of fracture during orthopedic clinic follow‐up visit post fracture admission. All BMD scans were performed on a single densitometer (Hologic QDR Discovery, Marlborough, MA, USA). The region of interest (ROI) was set as the total hip (non‐fractured hip site), femoral neck, and first to fourth lumbar vertebrae. We excluded vertebrae with fractures or degeneration causing >1 standard deviation greater areal BMD from the immediately adjacent vertebrae in accordance with the International Society for Clinical Densitometry guidelines for individual vertebrae exclusion. The BMD precision error (percentage of coefficient variation) was 1% for the total hip with a least significant change of 0.034 g/cm^2^, 2.3% for the femoral neck with a least significant change of 0.041 g/cm^2^, and 1% for the lumbar spine with a least significant change of 0.022 g/cm^2^. BMD results analyzed for the study were those that were documented within 6 months of the fracture event.

### 2.3 Statistical analysis

All statistical analysis was performed using STATA 16 (Stata Corporation, College Station, TX, USA). Data were expressed as mean ± standard deviation (SD) for numerical data or frequency (percentage) for categorical data. Binary logistic regression was used to assess univariate and multivariate regression analysis in demographic and clinical variables between patients with AFF and typical subtrochanteric femoral fractures. Variables that showed statistical significance (*p* < 0.05) in univariate analysis were included in the multivariate logistics regression to identify the independent factor that was associated with AFF risk.

### 2.4 Mortality

Patients were followed up until December 31, 2019, to assess their mortality rate. Date of death was accessed through the electronic health record, and whenever possible, the cause of death was recorded. We calculated Kaplan–Meier curves for death by categories of fracture types at 5 years. We then compared the risk of death between AFF and typical subtrochanteric femoral fractures with potential variables that may affect the progression of death in the follow‐up to assess both unadjusted and adjusted HR for death. This was done using Cox proportional hazard ratio to adjust for age, sex, eGFR status, DM2, race, and bisphosphonate used. To understand the excess mortality rate compared with the age‐standardized mortality of the general population, we compared the rate of death after an AFF and typical subtrochanteric femoral fractures to the rate of death in the general population. National death rates and age‐standardized death rates were accessed on the website from the department of statistics in Singapore (https://www.singstat.gov.sg/find-data/search-by-theme/population/death-and-life-expectancy/latest-data).

Standardized mortality ratios (SMR) were performed using STDRATE procedure in SAS University Edition (SAS Institute, Cary, NC, USA). A two‐tailed *p* < 0.05 was considered statistically significant. The study received approval by our institutional ethics board.

## 3 Results

**Fig 2:**
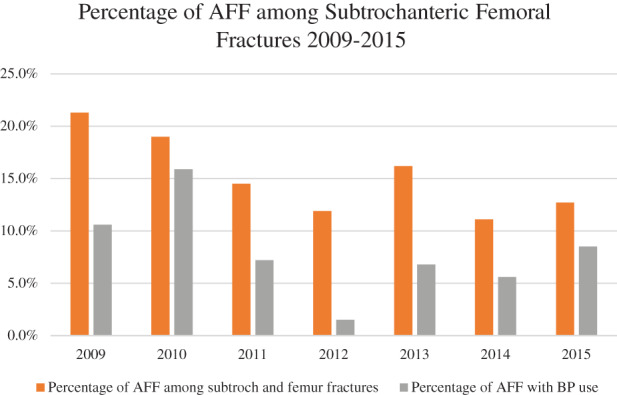
Percentage of atypical femoral fracture (AFF) among subtrochanteric femoral fractures from 2009 to 2015, with percentages of AFF with bisphosphonate (BP) use.

Sixty‐nine cases of AFF were identified by the ASBMR 2014 criteria; 5 cases had bilateral AFFs and 3 had incomplete fractures. AFF made up 2.0% of 3097 total hip, femur, and subtrochanteric fractures between 2009 and 2015. Patients presenting with bilateral AFFs were counted as a single AFF case for the purpose of this analysis. Indeterminate cases were excluded from this analysis. The percentage of AFF within subtrochanteric femur fractures in our population has also remained similar over 2009 to 2015 with the proportion of BP‐related AFF being stable (Fig. 2). The baseline demographic and clinical characteristics of the patients with subtrochanteric femoral fractures in the study are shown in Table 1. There were no statistically significant differences between the age of patients with AFF and typical subtrochanteric femoral fractures (71.2 versus 73.8 years old, *p* = 0.087). AFF patients had a lower mean Charlson comorbidity score (3.2 versus 3.9, *p* = 0.002) and lower prevalence of type 2 diabetes mellitus (13.0% versus 38.9%, *p* < 0.001) compared with typical subtrochanteric femoral fractures. There was a higher proportion of females in patients with AFF (87.0% versus 75.1%, *p* = 0.03), and there were no significant differences in the ethnicity of patients with AFF compared with the typical subtrochanteric femoral fractures. Data for 25‐OHD were only present for 76.4% (*n* = 353) of the cohort, TSH were recorded in 60% (*n* = 277), while among DM2 patients (*n* = 131), 80% had a recently documented HbA1C. There were no statistically significant differences between creatinine, eGFR, TSH, 25‐OHD, HbA1C, prevalence of smokers between patients with AFF, and typical subtrochanteric femoral fractures. There was no documented history of alcohol intake in any patients, and hence this data was excluded from the analysis. However, there was a higher rate of BP use (52.2% versus 11.2%, *p* < 0.001) and higher rate of surgical intervention (94.2% versus 73.9%, *p* < 0.001). There were no significant differences between prevalence of previous fragility fractures in the AFF and typical subtrochanteric femoral fracture group. There was no difference in the time to healing as documented by X‐ray, although there was higher prevalence of prodromal symptoms and bilateral fractures in AFF—this was not statistically significant. There were no differences in ethnicity groups between patients with AFF and typical subtrochanteric femoral fractures. Alendronate and risedronate were the most frequently prescribed BP; a small number of patients in both AFF and typical subtrochanteric groups had sequential treatments of oral BP (alendronate and risedronate) and sequential treatment to denosumab. Only one patient was exposed to zoledronic acid. Median duration of BP use was significantly longer in those with AFF (56.5 versus 15.5 months, *p* < 0.001). Glucocorticoid use was notably higher in patients with AFF (7.3 versus 1.3%, *p* = 0.002). Table 2 shows the BMD results of patients with AFF and typical subtrochanteric femoral fracture. It shows significantly higher BMD and *T*‐scores in total hip (0.70 g/cm^2^ versus 0.61 g/cm^2^, *p* = 0.008; −1.90 versus −2.73, *p* = 0.003) and femoral neck (0.61 g/cm^2^ versus 0.54 g/cm^2^, *p* = 0.028; −1.88 versus −2.54, *p* = 0.023) in patients with AFF versus those with typical subtrochanteric femoral factures. There were no significant differences in BMD of the lumbar spine.

**Table 1:** Demographics and Clinical Variables of Patients With Atypical Femoral Fracture (AFF) and Typical Subtrochanteric Femoral Fracture

	Atypical femoral fracture (*n* = 69)	Typical subtrochanteric femoral fracture (*n* = 393)	*p* Value
	*n* (%)	*n* (%)	
Age (years), mean (SD)	71.2 (8.7)	73.8 (12.3)	0.087
BMI (kg/m^2^), mean (SD)	23.5 (3.8)	23.9 (5.9)	0.754
Female	60 (87.0)	295 (75.1)	0.031
Race			
Chinese	51 (83.6)	266 (74.3)	0.144
Malay	5 (8.2)	66 (18.4)	
Indian/others	5 (8.2)	26 (7.3)	
eGFR mean (SD)	76.5 (21.5)	75.8 (35.1)	0.870
Creatinine median (IQR)	73 (62, 88)	77 (60, 100)	0.262
25OHD (μg/L), mean (SD) (*n* = 353)	28.6 (8.3)	22.3 (11.5)	0.061
Charlson comorbidity score, mean (SD)	3.2 (1.4)	3.9 (2.0)	0.002
Smokers	1 (1.5)	14 (3.7)	0.354
Fragility fracture history	15 (22.7)	105 (27.3)	0.433
Rheumatoid arthritis	5 (7.3)	4 (1.0)	0.001
Type 2 diabetes mellitus (DM2)	9 (13.0)	153 (38.9)	<0.001
HbA1c (%) in DM2, mean (SD) (*n* = 131)	6.52 (0.51)	7.25 (2.5)	0.257
TSH (mIU/L), median (IQR) (*n* = 277)	1.19 (0.56, 3.95)	1.50 (0.92, 2.50)	0.439
Prodromal symptoms	7 (10.1)	18 (4.6)	0.060
Bilateral fracture	3 (4.4)	10 (2.5)	0.403
Delayed healing	2 (2.9)	2 (2.3)	0.760
Surgical management	65 (94.2)	289 (73.9)	<0.001
Repeat surgical procedure	2 (2.9)	8 (2.1)	0.643
Time to healing (months), median (IQR)	2 (1, 3)	3 (1, 3)	0.480
Antiresorptive drug use (either oral BP/zoledronic acid/denosumab)	35 (50.7)	44 (11.2)	<0.001
Oral BP (either alendronate or risedronate)	28	35	
Oral BP sequential (alendronate and risedronate)	5	6	
Oral BP to denosumab	2	2	
Zoledronic acid	0	1	
Duration of BP use (months), median (IQR)	56.5 (28, 66)	15.5 (4, 36)	<0.001
Glucocorticoid use	5 (7.3)	5 (1.3)	0.002

SD = standard deviation; BMI = body mass index; eGFR = estimated glomerular filtration rate; IQR = interquartile range; 25‐OHD = 25‐hydroxyvitamin D; HbA1C = hemoglobin A1c; TSH = thyroid‐stimulating hormone; BP = bisphosphonate.

**Table 2:** Bone Mineral Density (BMD) and *T*‐Scores of Patients With Atypical Femoral Fracture and Typical Subtrochanteric Femoral Fracture

	Atypical femoral fracture (*n* = 69)	Typical subtrochanteric femoral fracture (*n* = 393)	*p* Value
BMD L spine, mean (SD)	0.79 (0.16)	0.78 (0.19)	0.791
BMD total hip, mean (SD)	0.70 (0.15)	0.61 (0.18)	0.008
BMD femoral neck, mean (SD)	0.61 (0.14)	0.54 (0.17)	0.028
*T‐*score L spine, mean (SD)	−1.58 (1.49)	−1.87 (1.57)	0.353
*T‐*score total hip, mean (SD)	−1.90 (1.35)	−2.73 (1.39)	0.003
*T‐*score femoral neck, mean (SD)	−1.88 (1.27)	−2.54 (1.45)	0.023

Multivariate regression analysis was performed to assess clinical variables that were significantly associated with AFF (Table 3) in patients presenting with subtrochanteric femoral fractures. There was a significant degree of collinearity between total hip *T*‐scores and FN *T*‐scores with similar odds ratio (OR) values observed in repeated regression. Creatinine and eGFR also yielded similar OR with significant degree of collinearity. Hence *T*‐score total hip and creatinine were selected for the final model. Exposure to bisphosphonate exhibits the strongest association with occurrence of AFF (OR = 6.65 [2.35–18.9], *p* < 0.001). A higher *T*‐score at the total hip (OR = 1.59 [1.06–2.39], *p* = 0.026) was also significantly associated with the incidence of AFF compared with typical subtrochanteric femoral fractures. Denosumab use was not significantly associated with AFF occurrence. A non‐DM2 status was also significantly associated with incidence of AFF compared with typical subtrochanteric femoral fractures.

**Table 3:** Multivariate Regression of Variables Associated With Atypical Femoral Fracture

	Unadjusted OR (95% CI)	Adjusted OR (95% CI)
Age (years)	0.98 (0.96, 1.00)^a^	1.00 (0.93, 1.07)
Female	2.21 (1.06, 4.63)^a^	1.94 (0.41, 9.16)
Creatinine	0.99 (0.98, 0.99)^a^	0.99 (0.98, 1.01)
Charlson comorbidity score	0.79 (0.68, 0.92)^a^	0.89 (0.57, 1.41)
Rheumatoid arthritis	7.58 (1.98, 28.97)^a^	0.29 (0.01, 6.59)
Type 2 diabetes mellitus	0.24 (0.11, 0.49)^a^	0.23 (0.06, 0.95)^a^
Bisphosphonate	8.17 (4.63, 14.4)^a^	6.65 (2.35, 18.9)^a^
Denosumab	5.84 (0.81, 42.1)	1.09 (0.05, 23.5)
Surgical management	5.74 (2.04, 16.1)^a^	5.91 (0.63, 55.7)
Glucocorticoid use	6.06 (1.71, 21.5)^a^	0.33 (0.03, 3.83)
T total hip	1.53 (1.14, 2.05)^a^	1.57 (1.04, 2.37)^a^

OR = odds ratio; CI = confidence interval.

^a^ *p* < 0.05.

**Fig 3:**
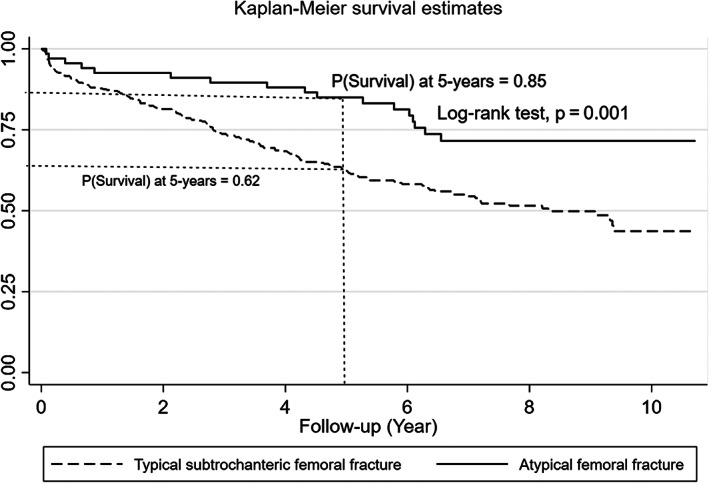
Kaplan–Meier curve for 5‐year survival in atypical femoral fracture versus typical subtrochanteric femoral fracture.

Mortality outcomes were assessed for patients with AFF versus typical subtrochanteric femoral fractures with censored date of December 31, 2019. There was a significant difference in mean survival time between AFF and typical subtrochanteric femoral fractures with probability of 5 years’ survival (0.85 versus 0.62, *p* = 0.001) (Fig. 3). In a multivariate analysis of factors associated with progression to death, older age, higher Charlson comorbidity score, and Malay ethnicity were most significantly associated with the highest risk of death (Table 4). The type of fracture (AFF or typical subtrochanteric femoral fracture) were not significantly associated with risk of death, implying that comorbidities of the patient rather than the fracture type were more important in the progression of death.

**Table 4:** Unadjusted and Adjusted Hazard Ratio (HR) of Progression to Death in Atypical and Typical Subtrochanteric Femoral Fractures

	Unadjusted HR (95% CI)	Adjusted HR (95% CI)
Atypical fracture	0.45 (0.27, 0.74)^a^	0.71 (0.40, 1.26)
Age	1.06 (1.05, 1.08)^a^	1.04 (1.02, 1.06)^a^
Female	1.48 (1.04, 2.11)^a^	0.92 (0.59, 1.44)
eGFR >60	0.54 (0.41, 0.72)^a^	0.87 (0.64, 1.20)
Charlson comorbidity score	1.53 (1.43, 1.63)^a^	1.44 (1.32, 1.58)^a^
Type 2 diabetes mellitus	1.17 (0.88, 1.56)	0.80 (0.57, 1.12)
Race		
Chinese	1.00	1.00
Malay	1.40 (0.98, 2.02)	1.50 (1.02, 2.22)^a^
Indian/other	0.95 (0.54, 1.69)	1.18 (0.63, 2.20)
Bisphosphonate	0.90 (0.62, 1.30)	1.13 (0.73, 1.76)

CI = confidence interval; eGFR = estimated glomerular filtration rate.

^a^ *p* < 0.05.

We compared standardized mortality rates of AFF patients to rates of death in the general population with similar age groups by dividing the patients and comparison groups into chronological 5‐year increments. We found that patients with AFF had a lower mortality rate (SMR = 0.66 [0.34–0.97], *p* = 0.03) compared with the national standardized age mortality rate, while those with typical subtrochanteric femoral fractures have an excess mortality rate compared with the national standardized age mortality rate (SMR = 6.80 [5.79–7.80], *p* < 0.001). To assess if there are subgroups within AFF and the typical subtrochanteric fractures that may have differing SMR, subgroup analyses were performed. SMR remained lower within subgroups of AFF patients stratified into sex, bisphosphonate use, and age. Within the group of patients with typical subtrochanteric femoral fractures, SMR remained higher in all subgroups except for men and BP users, which showed no differences in their SMR compared with the national standardized age mortality rate (Table 5).

**Table 5:** Age‐ and Sex‐Standardized Mortality Ratios (SMR) with 95% Confidence Intervals (CI) of Subgroups of Patients With Atypical Femoral Fracture and Typical Subtrochanteric Femoral Fracture

	SMR	95% CI	*p* Value
Atypical femoral fracture			
Any	0.66	0.34–0.97	0.030
Women	0.58	0.29–0.87	0.005
Men	0.08	0.00–0.18	<0.001
Bisphosphonate users	0.39	0.15–0.63	<0.001
Non‐bisphosphonate users	0.27	0.07–0.47	<0.001
Age <80 years	0.46	0.20–0.73	<0.001
Age ≥80 years	0.19	0.02–0.36	<0.001
Typical subtrochanteric femoral fracture			
Any	6.80	5.79–7.80	<0.001
Women	5.44	4.55–6.34	<0.001
Men	1.35	0.90–1.80	0.124
Bisphosphonate users	0.81	0.46–1.58	0.285
Non‐bisphosphonate users	5.98	5.04–6.93	<0.001
Age <80 years	3.17	2.48–3.85	<0.001
Age ≥80 years	3.63	2.90–4.36	<0.001

## 4 Discussion

Studies have shown that Asian patients are at higher risk of AFF, with a possibility of highest risk in Southeast Asian (SEA) patients.^(^ ^8^, ^9^ ^)^ In this study, we set out to assess demographic and clinical characteristics of AFF patients in our population. To our knowledge, this is the first study in the SEA population assessing clinical demographic characteristics and mortality post AFF. Our study found that AFF constitutes a small part of the overall cause of all hip and femoral fractures in our population at 2.0%. The percentage of AFF among subtrochanteric femoral fractures has remained stable from 2009 to 2015. The number of BP‐related AFF have also remained stable. However, the percentage of BP use in our population was very low during this time, as we have shown in a previous study the osteoporosis treatment gap in our population is very high, which points to the integral need of secondary fracture prevention.^(^ ^15^ ^)^ We also found, consistent with the current literature,^(^ ^6^, ^16^, ^17^ ^)^ that AFF patients are less likely to have concurrent comorbidity with a higher rate of glucocorticoid use. However, when we adjusted the odds ratio of AFF development considering all potential significant variables, only exposure to BP and absence of DM2 were associated with development of AFF. The lower incidence of DM2 in the AFF population was similarly reported in other studies.^(^ ^18^ ^)^ The strongest risk factor for development of AFF in our population was found to be exposure to bisphosphonate with a median duration of 56.5 months. This is consistent with a recent study that found increasing risk present at 3 to 5 years of BP exposure in the Asian population.^(^ ^8^ ^)^ However, our study also found that nearly half of AFF in our population were in patients not exposed to bisphosphonates; 47.8% of patients with AFF had no previously documented exposure to BP. This was similar to a previous Korean study that found 64.7% of their cohort with no previous BP exposure^(^ ^19^ ^)^ and past case series from Singapore,^(^ ^20^ ^)^ in contrast to studies in the ethnic white population that have shown that the majority of AFF occur in the context of BP exposure.^(^ ^21^ ^)^ It is also interesting to note that in our study a higher *T*‐score of the hip was associated with the incidence of AFF. This further supports the hypothesis that AFF is a separate category of fracture that resembles stress fractures, and a predisposition to these stress fractures in the Asian population even without exposure to BP may be related to geometrical differences and other factors discussed below. A stress fracture is thought to occur from excessive loading of a relatively healthy bone, whereas an insufficiency fracture occurs with normal loading of an abnormal or weakened bone.^(^ ^8^ ^)^ This may also explain why incidence of AFF was higher in the younger population with less comorbidity as they may be more mobile and engage in physical activity that may contribute to these stress fractures. BPs may potentially impact stress fracture healing and cause a propagation of microdamage and impending microcrack, which may progress to create a stress fracture. A growing number of studies support the association of femoral geometry, particularly greater femoral bowing and varus alignment, and AFFs.^(^ ^22^, ^23^, ^24^, ^25^ ^)^ These may be influenced by race or ethnicity and could explain the higher incidence of AFFs in the Asian population.^(^ ^8^ ^)^ It would also be important to consider other factors such as genetic predisposition with previous studies supporting the role of genetic influence of AFFs. Studies on whole‐exome sequencing in a family revealed a mutation in the enzymatic site inhibited by BP that may increase predisposition to AFFs.^(^ ^26^ ^)^ Other studies also support the presence of genetic variants that may be more common in the AFF patients with evidence suggesting that the risk may be polygenic with accumulation of at‐risk genetic variants.^(^ ^27^ ^)^ It would be important to study these variants in the Asian population to understand the underlying risk of AFF and how exposure to BP further increases this risk. Future studies are needed to better characterize underlying rates and physiology of AFF in both BP‐ and non‐BP‐exposed patients in these population as well.

We also sought to study the morbidity and mortality associated with AFF. Although surgical management in AFF patients is higher and there is a higher rate of bilateral fracture, our study found that time to healing and repeat surgery rates are not significantly different from typical fractures. In our follow‐up, we found that there was a higher probability of 5‐year survival for AFF patients compared with those with typical subtrochanteric femoral fractures (0.85 versus 0.62, *p* = 0.001). Factors associated with higher risk of progression to death were older age, higher Charlson comorbidity score, and Malay ethnicity. Indeed, when hazard ratios for death were adjusted for these variables at 10‐year follow‐up, the differences in mortality between AFF and typical subtrochanteric femoral fractures became non‐significant. This implies that comorbidities in patients presenting with these fractures are more important in predicting their mortality outcomes compared with the fracture type alone. The higher mortality rate in the Malay ethnic group compared with the Chinese is also consistent with observations from a previous hip registry study from Singapore.^(^ ^28^ ^)^ Other studies looking at mortality post AFF have shown no differences or reduced mortality compared with patients with typical subtrochanteric femoral fractures. A previous study in Sweden found lower mortality in patients with AFF in follow‐up duration of a mean of 4 years.^(^ ^12^ ^)^ Another study found no differences in mortality in a 30‐day follow‐up.^(^ ^13^ ^)^ When we compared the mortality rates of patients with AFF and typical subtrochanteric femoral fractures to the national standardized age mortality rate in the population in chronological 5‐year increments, we found that mortality risks for AFF patients were lower compared with the age‐standardized mortality in the general population; however, patients with typical fractures remain at a higher mortality risk compared with the general population. Data on comorbidities in the general population are not included in this comparison and thus the SMR should be interpreted in light of this. To assess the influence of factors such as sex, older age category, and bisphosphonate use, we performed further analysis to ascertain if there are differences in SMR in these subgroups. SMR remains lower in the AFF group compared with the age‐standardized mortality in the general population within the different subgroup analysis. In the typical subtrochanteric femoral fracture group, SMR remains higher compared with the age‐standardized mortality in the general population within the different subgroups except for men and those with BP use, which showed no significant difference in their mortality compared with the general population. This points to the fact that typical osteoporotic fracture such as the typical subtrochanteric femoral fracture remains a very high‐risk event when compared with the age‐standardized mortality rate. Bisphosphonates have been shown to reduce mortality post osteoporotic fracture^(^ ^29^, ^30^ ^)^ with increasing evidence of both skeletal and non‐skeletal pathways with benefits observed in cancer and cardiovascular outcomes.^(^ ^31^, ^32^, ^33^ ^)^ This may explain the lack of difference in the SMR in the typical subtrochanteric group compared with the general population in those who were BP users.

Taken together, these data imply that patients who sustain AFF are inherently different from those with typical subtrochanteric fracture. Despite higher rates of surgery within the AFF group, they show a higher 5‐year survival compared with patients with typical subtrochanteric fractures. Hazard ratio for progression to death shows that older age, comorbidities, and Malay ethnicity were the strongest predictors for death and that the type of fracture (AFF or typical subtrochanteric femoral fracture) was not significantly associated with progression to death. SMR of AFF is also lower than that of the age‐standardized mortality rate in the general population compared with typical subtrochanteric femoral fractures. There is also no evidence of increased morbidity or need for repeated surgical interventions. These data are reassuring for our population given the much higher risk of AFF that is known in the Asian population. Continued effort to reduce mortality in typical osteoporosis fractures should be pursued with the use of bisphosphonates and other anti‐osteoporotic drugs. The fear of AFF should not impede prevention of typical osteoporotic fractures.

The main strength of the study was long duration follow‐up of the patient and the link‐up of the electronic medical record for patients in Singapore, which allows us to accurately document a patient's clinical and medication history. We were able through this linkage to ascertain the date of death to provide accurate mortality data. We were also able to identify AFF by adjudication of the radiograph according to the ASBMR 2014 guideline. Limitations include the observational study design and the potential for residual confounding owing to differences in factors related to frailty, socioeconomic status, BMI, and BMD. There were missing data on 25‐OHD, TSH, and HbA1C, which may not accurately reflect differences present within the study population. There were very small numbers of patients on denosumab, and these patients were all previously exposed to BP. This may imply that we are unable to accurately ascertain the independent association of denosumab with AFF occurrence in this analysis. We also relied on the accuracy of the documented electronic health record for smoking and alcohol intake. There was no documented alcohol history intake in any patients, and we were unable to analyze this as a factor in contribution to the association with AFF and mortality. Ever‐smoker and current smokers were treated similarly in our analysis regardless of their last history of smoking. This may not accurately reflect the effect of smoking on the association of AFF and mortality as recency of exposure may play a significant part in the contribution of smoking as a pathological factor. We were also not fully able to ascertain the cause of death in most patients because the cause of death was not listed. There were also low numbers of patients in the older age groups and as such the interpretation of the results in these older groups should be done with caution. This study is also performed in a Southeast Asian population and as such its results may not be applicable in a different ethnicity setting. Comparison of the SMR with the general population is also limited by data on comorbidities present in the general population that was not adjusted for in the model.

In summary, AFF constitutes a small number of fractures in our population, and long‐term bisphosphonate exposure remains the strongest risk factor for its development. A total of 52.2% of AFF occur in BP users, and long‐term bisphosphonate use is the strongest risk factor for the incidence of AFF with median duration use of 56.5 months. Despite higher rates of surgery with AFF, there is no evidence of increased mortality or morbidity in this population compared with the typical subtrochanteric fracture. There was a lower risk of mortality in AFF compared with national age‐ and‐sex standardized mortality rate, but there was a higher mortality rate in the typical subtrochanteric fractures. The fear of AFF should not impede prevention of typical osteoporotic fractures, which carry a high risk of mortality. Further studies would be important to understand risk factors and physiology of the increased risk of AFF in the Asian population.

## Disclosures

All authors state that they have no conflict of interest.

## AUTHOR CONTRIBUTIONS

**Linsey Gani:** Conceptualization; data curation; formal analysis; investigation; methodology; project administration; validation; writing‐original draft; writing‐review & editing. **Le Roy Chong:** Data curation; investigation; validation; writing‐original draft; writing‐review & editing. **Natasha Anthony:** Data curation; project administration. **Lily Dacay:** Data curation; investigation; project administration. **Pei Tan:** Formal analysis; methodology; validation; writing‐original draft; writing‐review & editing. **Thomas King:** Conceptualization; formal analysis; investigation; methodology; project administration; supervision; validation; writing‐original draft; writing‐review & editing.

5

### PEER REVIEW

The peer review history for this article is available at https://publons.com/publon/10.1002/jbm4.10515.
